# Decreased α-cell mass and early structural alterations of the exocrine pancreas in patients with type 1 diabetes: An analysis based on the nPOD repository

**DOI:** 10.1371/journal.pone.0191528

**Published:** 2018-01-19

**Authors:** Fidéline Bonnet-Serrano, Marc Diedisheim, Roberto Mallone, Etienne Larger

**Affiliations:** 1 Assistance Publique Hôpitaux de Paris, Hôpital Cochin, UF d’Hormonologie, DHU « AUTHORS », Paris, France; 2 Assistance Publique Hôpitaux de Paris, Hôpital Cochin, Service de diabétologie, DHU « AUTHORS », Paris, France; 3 Université Paris Descartes, Sorbonne Paris Cité, Paris, France; 4 INSERM, U1016 and CNRS UMR8104, Cochin Institute, Paris, France; La Jolla Institute for Allergy and Immunology, UNITED STATES

## Abstract

**Background and aims:**

Abnormal glucagon secretion and functional alterations of the exocrine pancreas have been described in patients with type 1 diabetes (T1D), but their respective anatomical substrata have seldom been investigated. Our aim was to develop an automated morphometric analysis process to characterize the anatomy of α-cell and exocrine pancreas in patients with T1D, using the publicly available slides of the Network for Pancreatic Organ Donors (nPOD).

**Materials and methods:**

The ratio of β- and α-cell area to total tissue area were quantified in 75 patients with T1D (thereafter patients) and 66 control subjects (thereafter controls), on 2 insulin-stained and 4 glucagon-stained slides from both the head and the tail of the pancreas. The β- and α-cell masses were calculated in the 66 patients and the 50 controls for which the pancreas weight was available. Non-exocrine-non-endocrine tissue area (i.e. non-acinar, non-insular tissue) to total tissue area ratio was evaluated on both insulin- and glucagon-stained slides. Results were expressed as mean ±SD.

**Results:**

An automated quantification method was set up using the R software and was validated by quantification of β-cell mass, a well characterized parameter. β-cell mass was 29.6±112 mg in patients and 628 ±717 mg in controls (p<0.0001). α-cell mass was 181±176 mg in patients and 349 ±241mg in controls (p<0.0001). Non-exocrine-non-endocrine area to total tissue area ratio was 39±9% in patients and 29± 10% in controls (p<0.0001) and increased with age in both groups, with no correlation with diabetes duration in patients.

**Conclusion:**

The absolute α-cell mass was lower in patients compared to controls, in proportion to the decrease in pancreas weight observed in patients. Non-exocrine-non-endocrine area to total tissue area ratio increased with age in both groups but was higher in patients at all ages.

## Introduction

Type 1 diabetes (T1D) is the late consequence of a β-cell-targeted autoimmune disease that is thought to spare other endocrine and exocrine cell types of the pancreas. Functional alterations in glucagon secretion have long been described in T1D, including hyperglucagonemia, which may contribute to hyperglycemia [1–4] but also to decreased glucagon secretion in response to hypoglycemia [5,6]. However, there is little anatomical information on α-cell mass in T1D [7–10]. Similarly, the exocrine pancreas is both anatomically and functionally affected in long-standing T1D [11–15], but the causes of exocrine alterations are poorly understood. This raises the question whether the β-cell specific disease of T1D may later affect the pancreas as a whole.

The Network for Pancreatic Organ Donors (nPOD) has been created to provide access to high-quality pancreatic specimens for research purposes [16]. To date, the nPOD repository includes more than 400 pancreata. High-magnification digital images (virtual slides) stained for different markers are available on the nPOD website (www.jdrfnpod.org). Taking advantage of our previous experience with β-cell quantification [17], we developed an automated quantification method, using R software, to analyze the nPOD glucagon-stained virtual slides. We were thus able to characterize the α-cell mass in patients with T1D compared to non-diabetic control subjects and its relationship to disease duration.

## Design and methods

### Study subjects

Glucagon-stained digital slides of the pancreas from 112 patients with T1D and 114 non-diabetic control subjects were available from the nPOD repository as of April 2015. We elected to analyze only the 77 patients with T1D and 77 control donors for whom at least 4 glucagon-stained slides (2 from the head and 2 from the tail of the pancreas) and 2 insulin-stained slides (1 from the head and 1 from the tail) were available. Two patients with T1D were excluded because of slides that could not be used for quantification (poor quality of staining of section), and children under 4 years of age (n = 11 for control subjects and none for donors with T1D) were also excluded because of the reported specificities of the infant’s pancreas. Thus, we included 75 patients with T1D and 66 control subjects, for whom α-, β- exocrine and non-exocrine areas were measured. Non-exocrine-non-endocrine area was defined as the total pancreatic tissue area (excluding lymph nodes) minus the islet and the acinar area, thus corresponding to the sum of ducts, vessels, fatty degeneration and fibrosis. α- and β-cell mass were measurable only in the 66 patients with T1D and 50 control subjects for whom the weight of the pancreas was available. The whole process of slides selection has been summarized on a flowchart (S1 Fig). The nPOD project has been approved by the University of Florida Health Center Institutional Review Board. The nPOD scientific committee has been informed of the ongoing protocol. The present study was approved by the *Comité de Protection des Personnes Ile-de-France III (ref CCP AC043*, *23/02/2015)*.

### Pancreas sampling and processing

nPOD employs a standardized protocol for pancreas specimen preparation. Each pancreas is divided into three regions: the head, from the near-duodenal region up to the proximal isthmic region, located in front of the superior mesenteric vessels; the body and tail portions are then defined by dividing the remaining pancreatic tissue into two equal parts. Each region is processed into formalin-fixed paraffin blocks. Serial sections are stained by hematoxylin and dual-color immunohistochemistry, either Ki67 and insulin or CD3 and glucagon, visualized with peroxidase-diaminobenzidine and alkaline phosphatase-Fast Red polymer systems, respectively. Stained sections are scanned at 320x magnification using an Aperio CS scanner (Leica/Aperio) and pictures are finally stored in an online pathology database (eSLIDE; Leica/Aperio). For the present study, virtual slides were downloaded and analyzed at 100x magnification with a resolution of 1 μm/pixel, giving picture sizes comprised between 1x10^8^ and 6x10^8^ pixels.

### Statistical analysis

Data are expressed as mean (± SD). Analyses were carried out using R and GraphPad Prism 7. Differences between groups were evaluated using the Mann-Whitney non-parametric test. Correlations were evaluated using the Spearman signed rank test. Multivariate analysis with correlated variables was made by forward stepwise regression, the order of variables was determined by their explanatory power in univariate analysis.

## Results

### Development of an automated morphometric analysis process

α-cell area was quantified on glucagon-stained slides (4 slides by patient, 2 from the head and 2 from the tail), β-cell area on insulin-stained slides (2 slides by patient, 1 from the head and from the tail), and non-exocrine-non-endocrine (non-acinar, non-insular) pancreatic tissue area on both insulin- and glucagon-stained slides (i.e. 6 slides by patient, 3 from the head and 3 from the tail).

On each slide, three different areas were evaluated: the total pancreatic tissue area, the non-exocrine-non-endocrine pancreatic tissue area and the Fast Red-stained area, the latter corresponding either to alpha-cells or to beta-cells area, depending on the immunostaining.

In R software, a picture is converted into 3 matrices, corresponding respectively to the blue, green and red color levels, each matrix element accounting for one pixel. The principles used for the selection of the pixels, belonging to the 3 predefined areas were the following.

**Fig 1:**
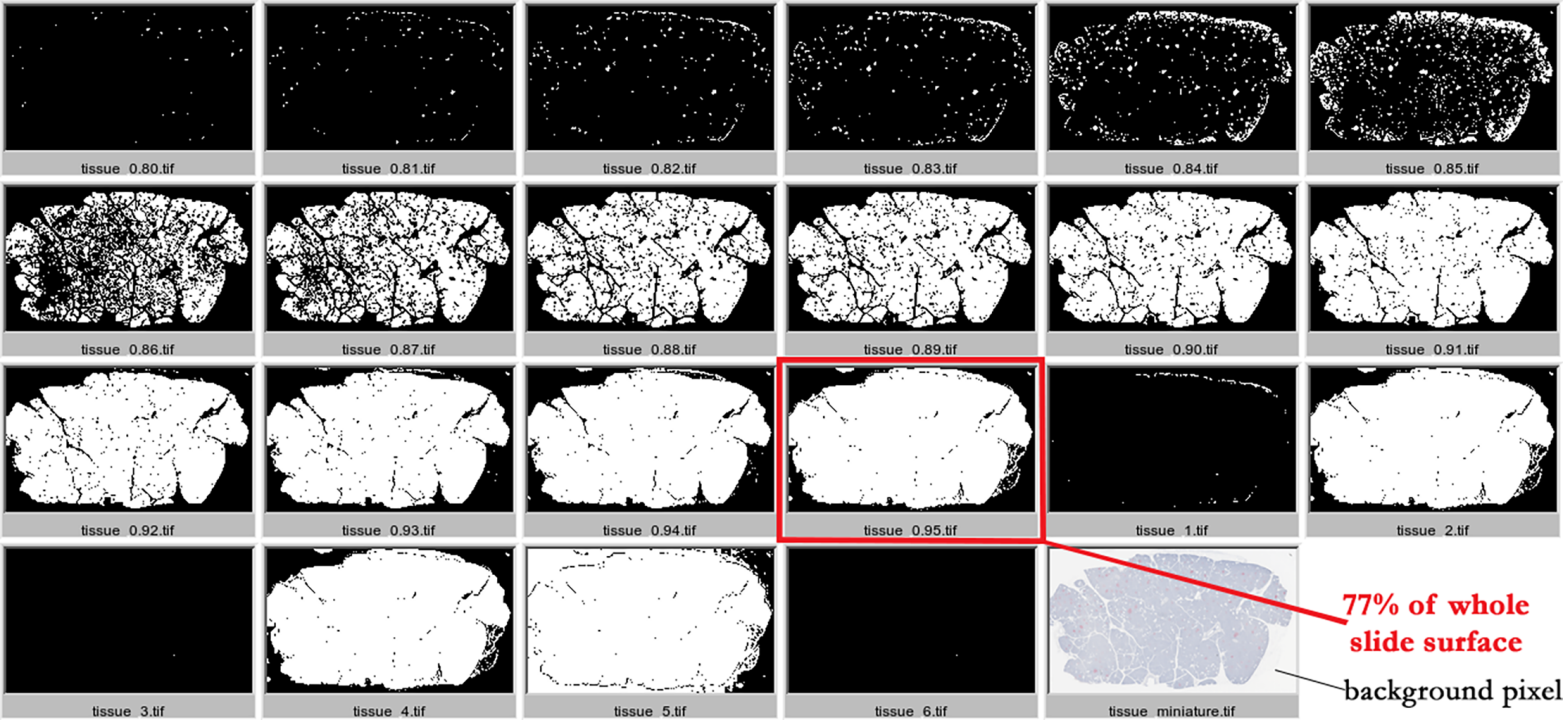
Example on the slide 14262. The first R script, used for the selection of the pixels belonging to total pancreatic tissue, generated 22 different propositions. The choice of the investigator is framed in red and the subsequent result indicated under the picture. An example of background pixel has been also pointed out on the picture miniature.

For total pancreatic tissue area delineation, the first step consisted in resizing the original picture to one hundredth of its full-resolution initial size. In other words, the original picture, whose size was between 1x10^8^ and 6x10^8^ pixels, was divided into squares of 10-pixel sides, each of these squares being integrated, in the resized picture, into one single central pixel, whose blue, green and red color levels were respectively the mean of blue, green and red levels from the 10x10 corresponding pixels in the original picture. This step allowed the smoothing of the existing discontinuities in the original picture to define a continuous pancreatic tissue surface (S2 Fig). The next step consisted then in the selection of any colored pixel, i.e. any pixel whose blue, green and red color levels were superior to the respective color levels of background pixels in the resized picture (Fig 1). The main difficulty in automating this process was to be able to take into account the differences in contrast and staining intensity between slides. The choice was therefore made to build an R script aimed at generating several propositions for total pancreatic tissue selection, by varying the color level threshold used by the different filters applied in the script (S1 File). An additional step of visual selection of the most accurate proposition by one investigator (FBS), in comparison to the original picture, was further carried out, allowing a critical step of visual control in the automated process (Fig 1).

**Fig 2:**
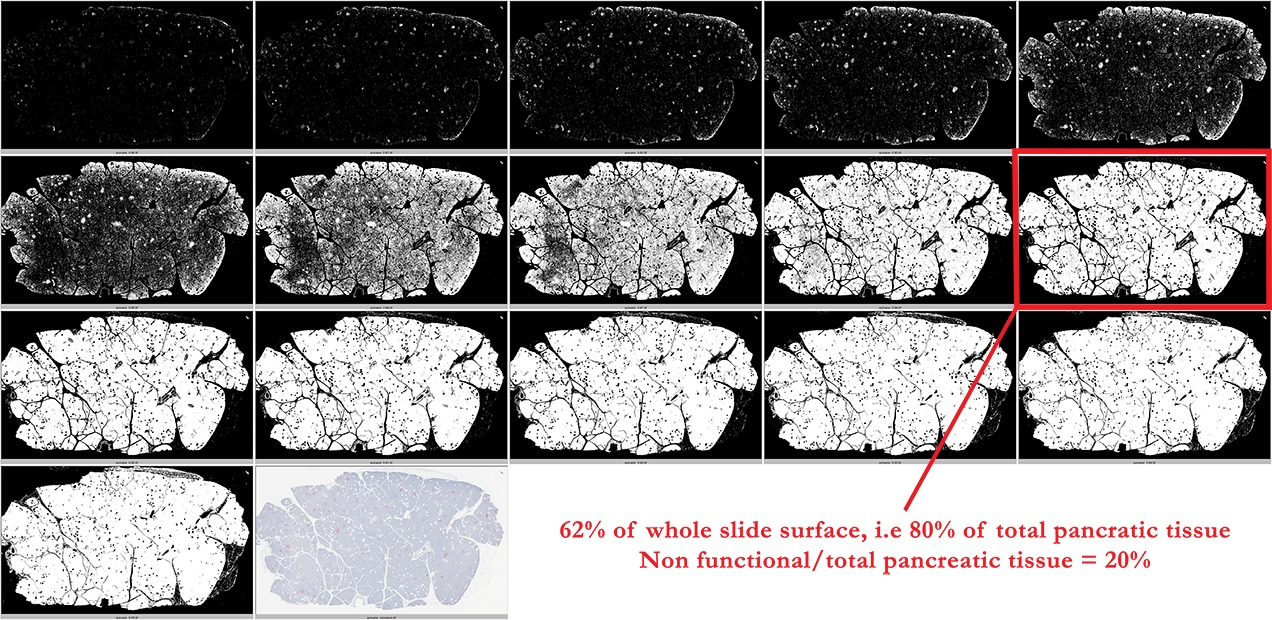
Example on the slide 14262. The second R script, used for the selection of the pixels belonging to functional pancreatic tissue, generated 16 different propositions. The choice of the investigator is framed in red and the subsequent results indicated under the picture.

For exocrine+endocrine pancreatic tissue area delineation, the first step also consisted in resizing the original picture, like in the total area delineation, but to a bigger size than for total pancreatic tissue, i.e. to one twentieth of its initial size. The next step consisted in the selection of either hematoxylin-stained blue pixels, i.e. colored pixels, whose blue level was superior to green and red levels, or to diaminobenzidine, brown, pixels or Fast Red, red, pixels, defined as colored pixels, whose red level was superior to green level. The R script intentionally generated automatically several propositions for exocrine+endocrine pancreatic tissue selection (S2 File). The choice of the most adequate selection by the investigator added a second step of visual control in the automated process (Fig 2).

**Fig 3:**
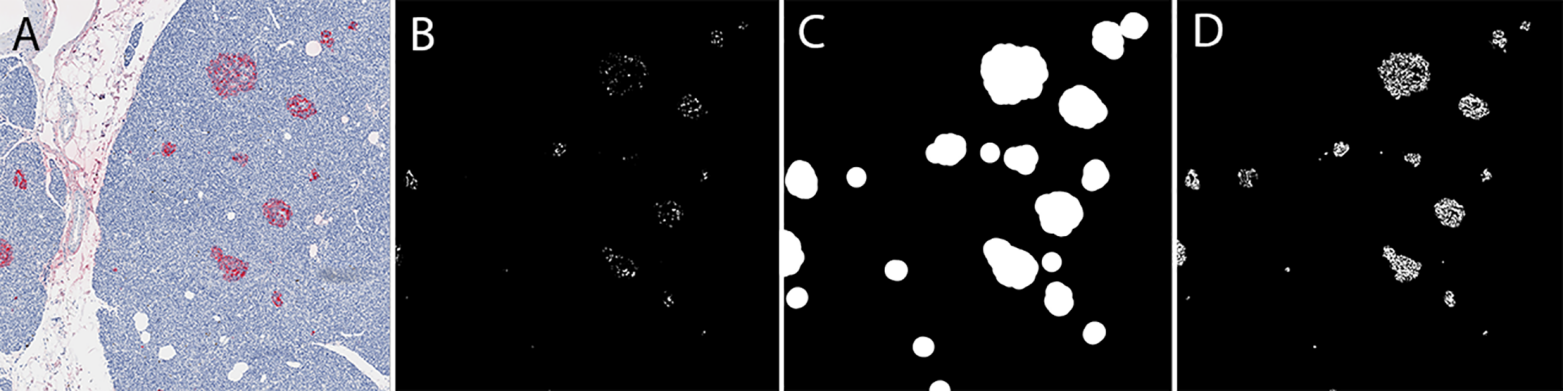
Representative example of the different steps used for selecting Fast Red-stained areas. A: Original image (slide 3160). B: Preselection of high-level red pixels. C: Definition of the regions of interest by circles of 50-pixel radius around each preselected pixel. D: The final output, as obtained by further selecting low-level red pixels in the regions of interest.

**Fig 4:**
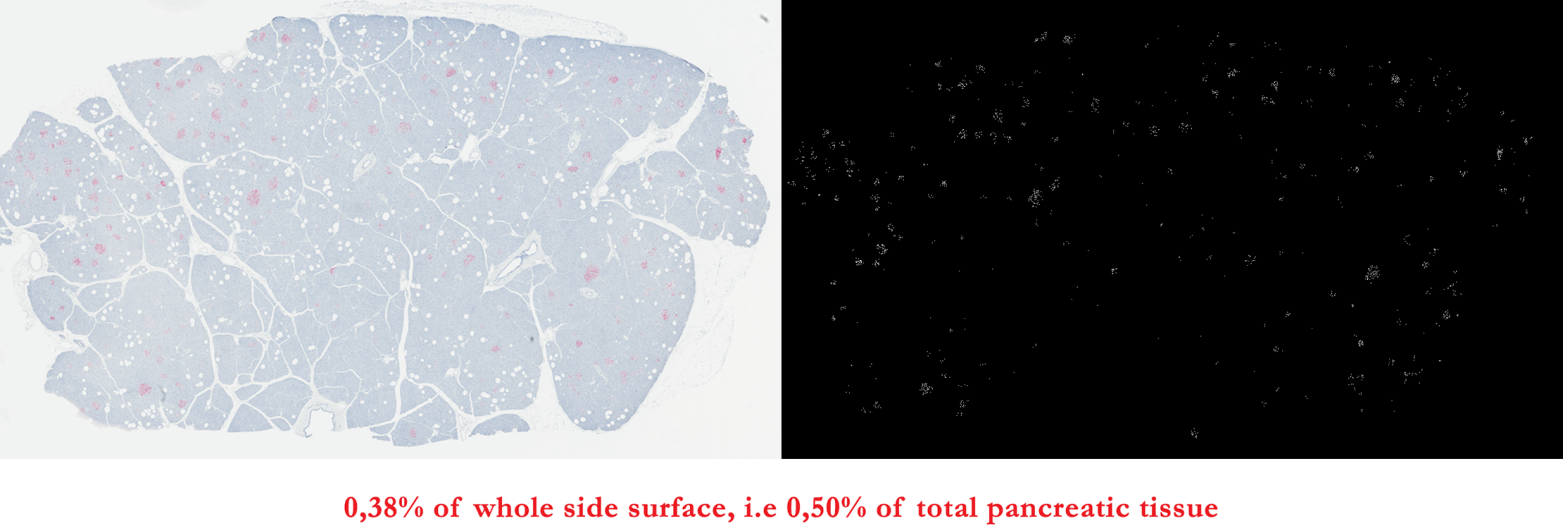
Example on the slide 14262. Selected Fast Red-stained pixels and subsequent results. Given that these 3 different areas were evaluated on pictures of different sizes (full-resolution picture for Fast Red-stained area versus resized pictures for total and exocrine+endocrine pancreatic tissue areas), all the results were normalized by the whole slide surface to allow further calculation of the parameters of interest, i.e. ratio of non-exocrine-non-endocrine pancreatic tissue (total pancreatic tissue minus exocrine+endocrine pancreatic tissue) to total pancreatic tissue area and ratio of either α-or β-cell area to total pancreatic tissue area, further converted into mass by multiplication by pancreas weight when this data was available. To take into account the difference in tissue area between slides, the average calculated for each patient, on respectively 6 slides for the ratio of non-exocrine-non-endocrine pancreatic to total pancreatic tissue, 4 slides for the ratio of α- cell area to total pancreatic tissue ratio and 2 slides for the ratio of β-cell area to total pancreatic tissue ratio, was weighted according to the total pancreatic tissue area of each slide.

Given the small size of Fast Red-stained regions, the use of the full resolution picture was necessary for appropriate selection of the corresponding area. However, given the large size of the original picture, this required its split into several smaller pictures to allow easy manipulations within R software. The final Fast Red-stained selected area was then calculated by summing the results obtained on each small picture. The selection principle of Fast Red-stained pixels relied on the selection of colored pixels whose red level was higher than blue and green levels. However, the main difficulty encountered in this process was the presence of light pink artifact pixels on some slides, most often organized into large very slightly colored sheets, but sometimes with a color intensity similar to that of some weakly Fast Red-stained alpha- or beta islet cells. Thus, to gain detection sensitivity without losing specificity, a multi-step selection R-script was built (Fig 3) (S3 File). Briefly, assumption was made that every truly positive Fast Red-stained pixel should either display a high red level itself or a high red level pixel in its vicinity; artifact pixels being low in intensity and located in expanded regions only containing low red level pixels. Hence, the first step of the script selected high red level pixels (Fig 3B); the second step drew circles with a 50-pixel radius around each selected pixel (Fig 3C), thus pinpointing the regions where true Fast Red-stained pixels were located; and the final step identified the red pixels with a lower threshold within these circles as Fast Red-stained pixels (Fig 3D). The main drawback of this approach should be the inappropriate selection of islet neighboring tissue submitted to bleeding of the overstaining existing on some slides. However, the validation of the present methodology on some well-established parameters, as beta-cell mass, suggested that this effect was negligible (Fig 4). The whole process of picture analysis has been summarized on a flowchart (S3 Fig) (S4 File).

### Characteristics of the subjects and of the pancreata

The clinical characteristics of subjects are summarized in Table 1, with the details of each studied case listed in S1 Table.

**Table 1:** Characteristics of study subjects and pancreata.

	Non diabetic subjects	Patients with T1D	p-value
	(N = 66)	(N = 75)	
**Gender ratio M/F (%)**	61/39	60/40	p = 0.94
**Age (years)**	30+-18	32+-19	p = 0.64
**BMI (kg/m^2^)**	26+-6	24+-4	p = 0.32
**HbA**_**1c**_ **(%)**	5.4+-1.3	9.7+-2.3	p<0.0001
**HbA**_**1c**_ **(mmol/mol)**	36+-9	83+-20	p<0.0001
**Pancreas weight (g)**	74+-23 (N = 50)	39+-16 (N = 66)	p<0.0001
**Diabetes duration (years)**		19.6+-18.2	

Data of many of the studied patients have already been presented in previous reports using the nPOD repository.

The weight of pancreas correlated with body weight both in patients with T1D (r = 0.53, p<0.0001, n = 66) and in control subjects (r = 0.63, p<0.0001, n = 50) (S4 Fig). However, as already reported for subjects of the nPOD repository [10,18] and elsewhere [11,12], the weight of the pancreas was significantly decreased in patients with T1D in whom it was 39 ±16 g as compared to 74 ±23 g in control subjects, (*p*<0.0001), suggesting that the whole pancreas is affected in T1D.

Furthermore, the weight of pancreas did not correlate with diabetes duration in patients with T1D (r = 0.10, p = 0.41, n = 66) (S5 Fig), suggesting that alterations of the whole pancreas occurred early in the natural story of disease.

### β-cell mass quantification validates the morphometric analysis method

**Fig 5:**
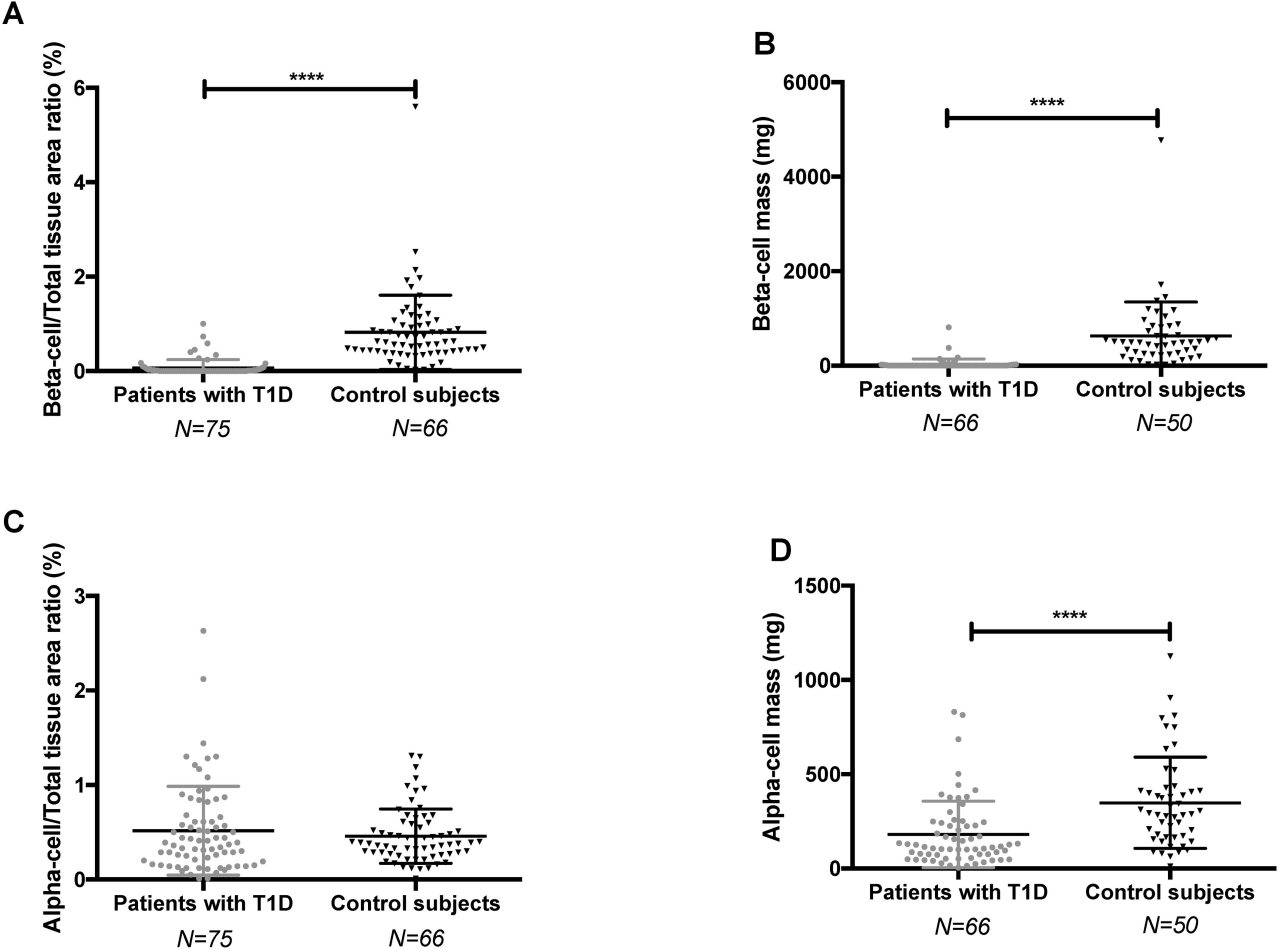
β- and α-cell to total tissue area ratio and β- and α-cell mass in patients with T1D and control subjects. A: β**-**cell area/total tissue area ratio. B: β**-**cell mass. T1D patients are symbolized by grey circles and controls by black triangles. Bars represent mean with SD and data were analyzed using the Mann-Whitney test. ****p<0.0001.

To validate our quantification method on Fast-red-stained slides, we first used it to quantify the β-cell mass, which is a well characterized parameter. Patients with T1D had a ratio of β-cell to total tissue area of 0.06±0.18% (n = 77), which was, as expected, significantly lower than that of control subjects in whom it was 0.82±0.78% (n = 66), (*p*<0.0001) (Fig 5A). Concordantly, the β-cell mass of patients with T1D was 29.6±112 mg (n = 66), and also drastically reduced compared to that of control subjects, in whom it was 628 ±717 mg (n = 50), (*p*<0.0001) (Fig 5B). The β-cell mass was however not correlated with duration of diabetes (r = -0.19, *p* = 0.13, n = 66) (S6 Fig). β-cell mass values here reported were similar to those reported in the literature, both in patients with T1D and in control subjects [10,17,19,20], thus validating our automated quantification method.

### The α-cell mass is lower but the relative α-cell area is similar in patients with T1D compared with controls

We quantified the α-cell area in patients with T1D and in control subjects and we calculated α-cell mass in the subjects in whom the weight of the pancreas was available. The ratio of α-cell to total tissue area ratio was slightly but not significantly higher in patients with T1D in whom it was 0.52±0.47% (n = 75) as compared to 0.46±0.29% (n = 66) in control subjects (*p* = 0.77; Fig 5C). However, due to the lower weight of the pancreas in patients with T1D, the α-cell mass of the patients with T1D, 181±176 mg (n = 66) was significantly lower than that of control subjects, in whom it was 349±241 mg (n = 50, *p*<0.0001; Fig 5D).

**Fig 6:**
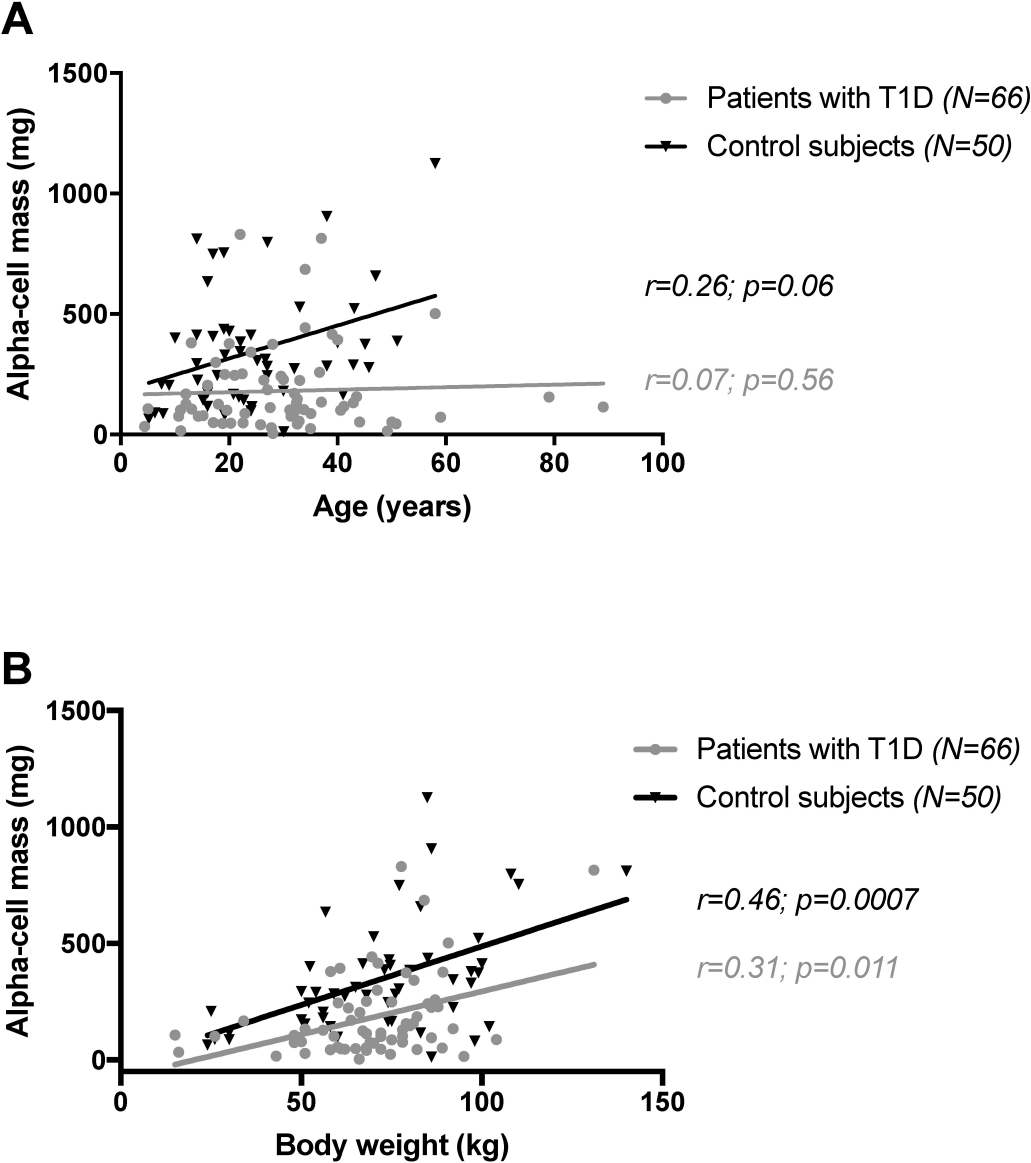
**A: Correlation between α-cell mass and age in patients with T1D and control subjects**. **B: Correlation between α-cell mass and body weight in patients with T1D and control subjects.** T1D patients are symbolized by grey circles and controls by black triangles.

The α-cell mass did not correlate with age, neither in patients with T1D (r = 0.07, *p* = 0.56 n = 66), nor in control subjects (r = 0.26, p = 0.06, n = 50). However, there was a trend toward increased α-cell mass with age in controls, which was not observed in patients with T1D (Fig 6A). α-cell mass correlated with body weight both in patients with T1D (r = 0.31, *p* = 0.011, n = 66) and in control subjects *(*r = 0.46, *p* = 0.0007, n = 50) (Fig 6B). Finally, α-cell mass did not correlate with disease duration (r = -0.03, *p* = 0.83, n = 66) in patients with T1D (S7 Fig).

**Fig 7:**
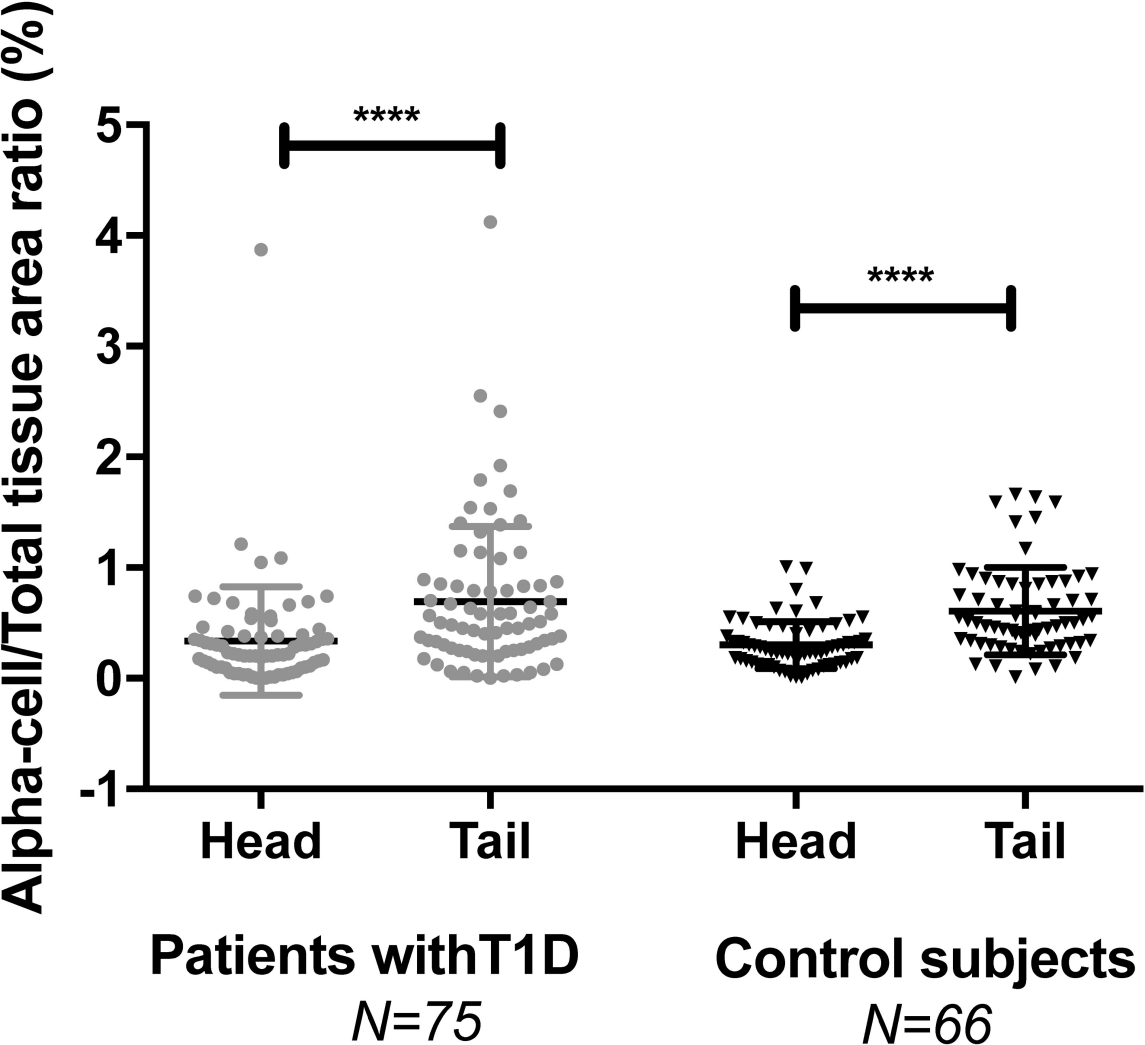
α-cell to total tissue area ratio in the head and tail of the pancreas of patients with T1D and control subjects. Data representation is the same as in Fig 5.

We next investigated the distribution of α-cells throughout the pancreas. Both in T1D patients and controls, the α-cell/total tissue area ratio was significantly lower in the head compared with the tail of the pancreas (0.34% in the head vs 0.69% in the tail in T1D patients and 0.30% in the head vs. 0.61% in the tail in controls, p<0.0001 in both cases; Fig 7).

Thus, while the α-cell/total tissue area ratio and the distribution of α-cells within the pancreas were not different between T1D patients and controls, the α-cell mass was lower in T1D patients, reflecting the decreased weight of the pancreas.

### Non-exocrine-non-endocrine pancreatic tissue, i. e., fatty degeneration and fibrosis in T1D

**Fig 8:**
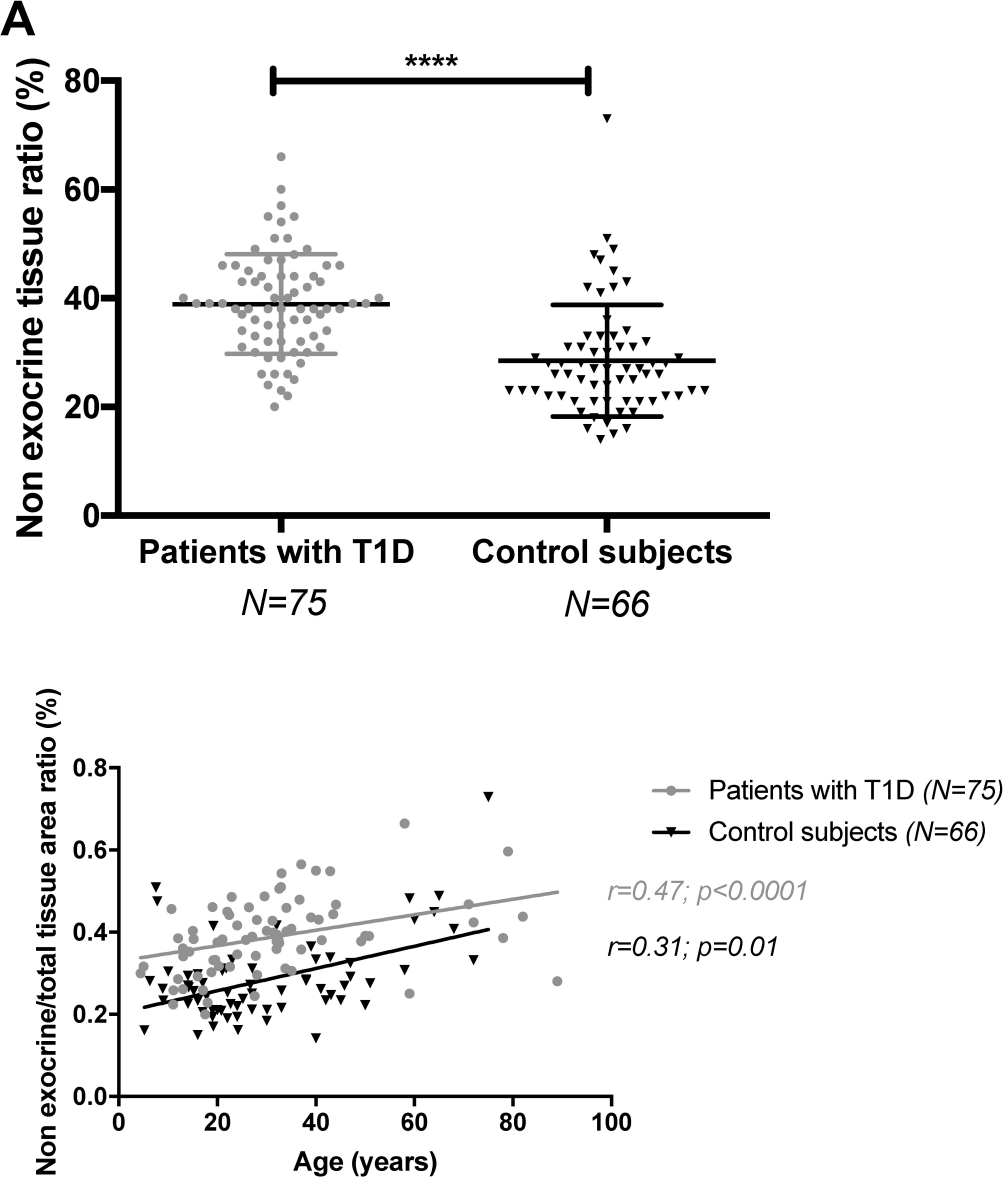
**Non-exocrine-non-endocrine tissue area/total tissue area ratio in patients with T1D and control subjects (A) and its correlation with age (B).** Data representation is the same as in Figs 5 and 6, respectively.

Because the weight of the pancreas was decreased in patients with T1D, who also had decreased β- and α-cell mass, and because alterations of the exocrine function have been described in long term in patients with T1D [14], we asked whether there was an anatomical substratum to altered exocrine function in T1D. Structural alterations of the exocrine pancreas were expressed as the ratio of non-exocrine-non endocrine tissue area to total pancreas area [17]. This ratio was higher in patients with T1D (39±9%, n = 75) than in control subjects (29±10%, n = 66, *p*<0.0001; Fig 8A). The value we observed in control subjects was very similar to that we observed in a previous analysis of control subjects of the nPOD repository [17]. The ratio of non-exocrine-non-endocrine tissue area to total pancreas area correlated with age in both groups: r = 0.47 in patients with T1D, (n = 75, *p*<0.0001) and r = 0.31 in control subjects (n = 66, *p* = 0.01). Nevertheless, patients with T1D had higher ratio of non-exocrine-non-endocrine to total pancreas area than control subjects at all ages (Fig 8B).

Non-exocrine non-endocrine to total pancreas area ratio was weakly correlated with diabetes duration in patients with T1D (r = 0.26, *p* = 0.02, n = 75) (S8 Fig). However, this association was not significant in multivariate analysis that included both age and diabetes duration as variables.

These results show that structural alterations, fatty degeneration and fibrosis, of the exocrine pancreas increased with age both in patients with T1D and control subjects, and that these structural alterations were more severe in patients, irrespective of T1D duration. Collectively, these results suggest that T1D does not only affect β-cell but also impairs α-cells and the exocrine pancreas.

## Discussion

Despite well characterized alterations in glucagon secretion and exocrine function in patients with T1D, there are few anatomical descriptions that could help understanding these functional alterations. By describing such anatomical alterations, we were interested to know whether anatomical alterations of α-cells and exocrine pancreas were associated in patients with T1D, a fact that would suggest that the disease may extend beyond β-cells. The nPOD project offered a good opportunity for such a study. Building on our previous report about β-cell quantification [17], we here developed a similar strategy to quantify α-cells in patients with T1D. Our method was extensively automated yet open to visual inspection at each step, which is rarely the case in other quantification studies. Another advantage that may encourage further studies on this and other repositories is the use of the free software R as a single platform for both image analysis and surface ratio calculations. The quantification method was first validated for β-cell mass assessment, a parameter that has been extensively studied, including in the nPOD repository [10,17]. In non-diabetic control subjects, β-cell mass was close to that calculated in nPOD subjects, by us [17] and by others [10]. Elsewhere, the β-cell mass was 800–900 mg in adults [19,20]. The present method thus gave slightly lower estimates than the literature, at least in part because, unlike methods based on point counting, we did not take into account the nuclear area, since only the cytoplasm was Fast-red-stained. In patients with T1D, the β-cell mass was close to zero, confirming our previous study [17] and another nPOD-based study [10].

The main finding of the present work was that the α-cell mass was decreased in patients with T1D. The α-cell mass has seldom been measured in patients with either T1D [7–10] or type 2 diabetes [8,21,22]. In control subjects, α-cell mass was previously measured at 347±183 mg (n = 52) [21], very close to our calculated value at 349±241 mg (n = 66). In comparison, Campbell-Thompson reported a higher value of 495±320 mg (n = 57) in control subjects [10]. The ratio of α-cell area to pancreas area was previously estimated at 0.49±0.44% [22], also close to our own estimation and slightly diverging from another estimation based on nPOD data, in which the α-cell area ratio was found to be ~0.7% in non-diabetic subjects [10]. Based on these latter findings, Campbell-Thompson et al. [10] reported that the α-cell mass was similar in patients with T1D and in control subjects, whereas we report that the α-cell mass was decreased in patients with T1D. To our knowledge, the study of J. Rahier et al. [8] was the only other study that quantified α-cell mass in subjects with T1D. These authors reported that the α-cell mass was not significantly lower in patients with T1D, but their study was underpowered due to the analysis of only 4 pancreata from patients with T1D and 8 organs from control subjects [8]. Thus, although the present quantification of α-cell mass differs from that of Campbell-Thompson et al. [10], our results in control subjects are close to other published data, thus further validating our method.

The correlation between α-cell mass and body weight we observed in control subjects is consistent with the recently described increased fasting plasma glucagon concentration observed in subjects with insulin resistance [23]. However, whether decreased α-cell mass in T1D has functional implications is difficult to appreciate in light of current knowledge, considering the complexity of regulation of glucagon secretion. Indeed, patients with T1D have been reported to harbor functional alterations in glucagon secretion that go in two opposite directions, i. e. decreased glucagon secretion in response to hypoglycemia [5,6], and fasting hyperglucagonemia with inadequate plasma glucagon decrease in response to meals [2,3,24]. Inadequate glucagon suppression after meals is observed at the time of T1D diagnosis, and worsens during the first following years [25]. Nevertheless, the relative hyperglucagonemia is not inconsistent with our result of decreased α-cell mass in T1D patients. Indeed, the reduction we observed in α-cell mass was much lower than the reduction in β-cell mass. Furthermore, it is now established that there are extra-pancreatic sources of glucagon in man [26] that respond paradoxically to oral challenge [27], making the identification of the source of circulating postprandial glucagon currently unsettled in man.

However, the link between beta-cell mass and beta-cell function has been previously established in several studies in healthy living donors who had undergone hemipancreatectomy [28–30], all of them reporting a deterioration of insulin secretion and glucose tolerance in these subjects. This model is particularly relevant since individuals undergoing hemipancreatectomy exhibit a significant decrease in beta cell mass while beta-cell area to total pancreatic tissue ratio is conserved. Moreover, Seaquist and al. [31] also reported that alpha cell function was altered in healthy living donors who had undergone hemipancreatectomy, as shown by a decrease in arginine-induced glucagon secretion in comparison to a population of matched controls.

The paradoxical decrease in α-cell mass with preserved α-cell ratio in patients with T1D reflected the decrease of pancreas weight [11–13]. This confirmed that the exocrine tissue is also affected in T1D, since endocrine tissues represent only 1–2% of the total pancreatic mass. In addition to the decrease in pancreas weight, there were structural alterations of the exocrine tissue in patients with T1D, that, at variance with the functional alterations of exocrine function, were not associated with T1D duration [13,14]. As previously described [17], non-exocrine-non-endocrine tissue increased significantly with age, both in patients with T1D and in control subjects, reflecting the age-associated fatty degeneration of the pancreas [32]. However, patients with T1D had a higher non-exocrine-non-endocrine tissue area to total pancreas area than non-diabetic subjects regardless of age, suggesting that T1D is a disease not confined to β-cells but rather involving the whole pancreas. Infiltration of the exocrine pancreas by CD8+ T cells is common observation in patients with type 1 diabetes [33] and O Korsgren has made the hypothesis that type 1 diabetes is mainly a disease of the whole pancreas [34]. However, contrary to β-cells, α-cells did not seem to be specifically targeted in T1D, since their decrease paralleled that of the whole pancreas, while the α-cell to total pancreas area ratio was unchanged compared to control subjects.

In conclusion, based on a novel quantification method of nPOD virtual slides, we observed that the α-cell mass was reduced in patients with T1D, while the ratio of α-cell to pancreas area was not affected. Pancreas fatty degeneration was associated with ageing in both control subjects and patients with T1D but was higher in patients with T1D at all ages and irrespective of T1D duration, pointing to an early event in the natural history of the disease. Together with the reported decrease in pancreas weight, these results collectively suggest that T1D is not limited to β-cells, but is associated with structural alterations of both the α-cells and the exocrine pancreas.

## Supporting information

S1 Fig Process of slides selection. (TIF)

S2 Fig Resizing the original picture to one hundredth of its full-resolution initial size allowed the smoothing of tissue discontinuities. (TIF)

S3 Fig Whole process of image analysis. (TIF)

S4 Fig Correlation between pancreas weight and body weight in patients with T1D and control subjects. Data representation is the same as in Fig 6.
(TIF)

S5 Fig Correlation between pancreas weight and diabetes duration in patients with T1D. Data representation is the same as in Fig 6.
(TIF)

S6 Fig Correlation between β-cell mass and diabetes duration in patients with T1D. Data representation is the same as in Fig 6.
(TIF)

S7 Fig Correlation between α-cell mass and T1D duration. Data representation is the same as in Fig 6.
(TIF)

S8 Fig Correlation between non-exocrine tissue area/total tissue area ratio and T1D duration. Data representation is the same as in Fig 6.
(TIF)

S1 Table Detailed characteristics of study subjects and pancreata. (XLSX)

S1 File R-script used for total pancreatic tissue detection. (R)

S2 File R-script used for exocrine+endocrine pancreatic tissue detection. (R)

S3 File R-script used for Fast Red-stained area detection. (R)

S4 File R-script used for area quantification. (R)

## References

[1] Cryer PE. Minireview: Glucagon in the pathogenesis of hypoglycemia and hyperglycemia in diabetes. Endocrinology. 2012;153: 1039–48. doi: 10.1210/en.2011-1499 22166985 10.1210/en.2011-1499 PMC3281526

[2] Fredheim S, Andersen M-LM, Pörksen S, Nielsen LB, Pipper C, Hansen L, et al The influence of glucagon on postprandial hyperglycaemia in children 5 years after onset of type 1 diabetes. Diabetologia. 2015;58: 828–34. doi: 10.1007/s00125-014-3486-3 25541633 10.1007/s00125-014-3486-3

[3] Kramer CK, Borgoño CA, Van Nostrand P, Retnakaran R, Zinman B. Glucagon response to oral glucose challenge in type 1 diabetes: lack of impact of euglycemia. Diabetes Care. 2014;37: 1076–82. doi: 10.2337/dc13-2339 24241790 10.2337/dc13-2339

[4] Unger RH, Cherrington AD. Glucagonocentric restructuring of diabetes: a pathophysiologic and therapeutic makeover. J Clin Invest. 2012;122: 4–12. doi: 10.1172/JCI60016 22214853 10.1172/JCI60016 PMC3248306

[5] Gerich JE, Langlois M, Noacco C, Karam JH. Lack of Glucagon Response to Hypoglycemia in Diabetes: Evidence for an Intrinsic Pancreatic Alpha Cell Defect. Forsham Source Sci New Ser. 1973;182: 171–173. Available: http://www.jstor.org/stable/1736097 10.1126/science.182.4108.171 4581053

[6] Banarer S, McGregor VP, Cryer PE. Intraislet hyperinsulinemia prevents the glucagon response to hypoglycemia despite an intact autonomic response. Diabetes. 2002;51: 958–65. Available: http://www.ncbi.nlm.nih.gov/pubmed/11916913 11916913 10.2337/diabetes.51.4.958

[7] Orci L, Baetens D, Rufener C, Amherdt M, Ravazzola M, Studer P, et al Hypertrophy and hyperplasia of somatostatin-containing D-cells in diabetes. Proc Natl Acad Sci U S A. 1976;73: 1338–42. Available: http://www.ncbi.nlm.nih.gov/pubmed/131313 131313 10.1073/pnas.73.4.1338 PMC430269

[8] Rahier J, Goebbels RM, Henquin JC. Cellular composition of the human diabetic pancreas. Diabetologia. 1983;24: 366–71. doi: 10.1007/BF00251826 6347784 10.1007/BF00251826

[9] Waguri M, Hanafusa T, Itoh N, Miyagawa J-I, Imagawa A, Kuwajima M, et al Histopathologic Study of Lymphocytic Infiltration the Pancreas Shows in Japanese Patients a Characterist with IDDM. Endocr J. 1997;44: 23–33. 9152611 10.1507/endocrj.44.23

[10] Campbell-Thompson M, Fu A, Kaddis JS, Wasserfall C, Schatz DA, Pugliese A, et al Insulitis and β-Cell Mass in the Natural History of Type 1 Diabetes. Diabetes. 2016;65: 719–731. doi: 10.2337/db15-0779 26581594 10.2337/db15-0779 PMC4764143

[11] Altobelli E, Blasetti a, Verrotti a, Di Giandomenico V, Bonomo L, Chiarelli F. Size of pancreas in children and adolescents with type I (insulin-dependent) diabetes. J Clin Ultrasound. 1998;26: 391–395. 9783245 10.1002/(sici)1097-0096(199810)26:8<391::aid-jcu3>3.0.co;2-d

[12] Fonseca V, Berger L a, Beckett a G, Dandona P. Size of pancreas in diabetes mellitus: a study based on ultrasound. Br Med J (Clin Res Ed). 1985;291: 1240–1. doi: 10.1136/bmj.291.6504.1240 10.1136/bmj.291.6504.1240 PMC1417112 3933616

[13] Philippe M-F, Benabadji S, Barbot-Trystram L, Vadrot D, Boitard C, Larger E. Pancreatic volume and endocrine and exocrine functions in patients with diabetes. Pancreas. United States; 2011;40: 359–363. doi: 10.1097/MPA.0b013e3182072032 21283038 10.1097/MPA.0b013e3182072032

[14] Larger E, Philippe MF, Barbot-Trystram L, Radu A, Rotariu M, Nobecourt E, et al Pancreatic exocrine function in patients with diabetes. Diabet Med. England; 2012;29: 1047–1054. doi: 10.1111/j.1464-5491.2012.03597.x 22273174 10.1111/j.1464-5491.2012.03597.x

[15] Gaglia JL, Guimaraes AR, Harisinghani M, Turvey SE, Jackson R, Benoist C, et al Brief report Noninvasive imaging of pancreatic islet inflammation in type 1A diabetes patients. J Clin Invest. 2011;121: 5–8. doi: 10.1172/JCI44339DS1 10.1172/JCI44339 PMC3007157 21123946

[16] Campbell-Thompson M, Wasserfall C, Kaddis J, Albanese-O’Neill A, Staeva T, Nierras C, et al Network for Pancreatic Organ Donors with Diabetes (nPOD): Developing a tissue biobank for type 1 diabetes. Diabetes Metab Res Rev. 2012;28: 608–617. doi: 10.1002/dmrr.2316 22585677 10.1002/dmrr.2316 PMC3456997

[17] Diedisheim M, Mallone R, Boitard C, Larger E. ?-cell Mass in Nondiabetic Autoantibody-Positive Subjects: An Analysis Based on the Network for Pancreatic Organ Donors Database. J Clin Endocrinol Metab. 2016;101: 1390–1397c. doi: 10.1210/jc.2015-3756 26829442 10.1210/jc.2015-3756

[18] Campbell-Thompson ML, Kaddis JS, Wasserfall C, Haller MJ, Pugliese A, Schatz DA, et al The influence of type 1 diabetes on pancreatic weight. Diabetologia. 2016;59: 217–221. doi: 10.1007/s00125-015-3752-z 26358584 10.1007/s00125-015-3752-z PMC4670792

[19] Rahier J, Guiot Y, Goebbels RM, Sempoux C, Henquin JC. Pancreatic ?-cell mass in European subjects with type 2 diabetes. Diabetes, Obes Metab. 2008;10: 32–42. doi: 10.1111/j.1463-1326.2008.00969.x 18834431 10.1111/j.1463-1326.2008.00969.x

[20] Saisho Y, Butler AE, Manesso E, Elashoff D, Rizza R a., Butler PC. Beta-Cell mass and turnover in humans: Effects of obesity and aging. Diabetes Care. 2013;36: 111–117. doi: 10.2337/dc12-0421 22875233 10.2337/dc12-0421 PMC3526241

[21] Henquin JC, Rahier J. Pancreatic alpha cell mass in European subjects with type 2 diabetes. Diabetologia. 2011;54: 1720–5. doi: 10.1007/s00125-011-2118-4 21465328 10.1007/s00125-011-2118-4 PMC3110273

[22] Inaishi J, Saisho Y, Sato S, Kou K, Murakami R, Watanabe Y, et al Effects of obesity and diabetes on alpha and beta cell mass in surgically resected human pancreas. J Clin Endocrinol Metab. 2016; jc.2016-1374. doi: 10.1210/jc.2016-1374 27070277 10.1210/jc.2016-1374 PMC4929842

[23] Faerch K, Vistisen D, Pacini G, Torekov SS, Johansen NB, Witte DR, et al Insulin Resistance is Accompanied by Increased Fasting Glucagon and Delayed Glucagon Suppression in Individuals With Normal and Impaired Glucose Regulation. Diabetes. 2016; doi: 10.2337/db16-0240 27504013 10.2337/db16-0240

[24] Unger RH, Aguilar-Parada E, Müller WA, Eisentraut AM. Studies of pancreatic alpha cell function in normal and diabetic subjects. J Clin Invest. 1970;49: 837–48. doi: 10.1172/JCI106297 4986215 10.1172/JCI106297 PMC322540

[25] Brown RJ, Slnaii N, Rother KI. Too much glucagon, too little insulin: Time course of pancreatic islet dysfunction in new-onset type 1 diabetes. Diabetes Care. 2008;31: 1403–1404. doi: 10.2337/dc08-0575 18594062 10.2337/dc08-0575 PMC2453684

[26] Ravazzola M, Unger RH, Orci L. Demonstration of glucagon in the stomach of human fetuses. Diabetes. 1981;30: 879–82. Available: http://www.ncbi.nlm.nih.gov/pubmed/7024024 7024024 10.2337/diab.30.10.879

[27] Lund A, Bagger JI, Wewer Albrechtsen NJ, Christensen M, Grondahl M, Hartmann B, et al Evidence of Extrapancreatic Glucagon Secretion in Man. Diabetes. United States; 2016;65: 585–597. doi: 10.2337/db15-1541 26672094 10.2337/db15-1541

[28] Kendall DM, Sutherland DER, Najarian JS, Goetz FC, Robertson RP. Effects of Hemipancreatectomy on Insulin Secretion and Glucose Tolerance in Healthy Humans. N Engl J Med. 1990;322: 898–903. doi: 10.1056/NEJM199003293221305 2179721 10.1056/NEJM199003293221305

[29] Kumar AF, Gruessner RWG, Seaquist ER. Risk of glucose intolerance and diabetes in hemipancreatectomized donors selected for normal preoperative glucose metabolism. Diabetes Care. 2008;31: 1639–1643. doi: 10.2337/dc07-2453 18469205 10.2337/dc07-2453 PMC2494634

[30] Lam NN, Schnitzler MA, Segev DL, Hess GP, Kasiske BL, Randall HB, et al Diabetes Mellitus in Living Pancreas Donors: Use of Integrated National Registry and Pharmacy Claims Data to Characterize Donation-Related Health Outcomes. Transplantation. United States; 2017;101: 1276–1281. doi: 10.1097/TP.0000000000001375 27482962 10.1097/TP.0000000000001375 PMC5288378

[31] Seaquist ER, Robertson RP. Effects of hemipancreatectomy on pancreatic alpha and beta cell function in healthy human donors. J Clin Invest. 1992;89: 1761–6. doi: 10.1172/JCI115779 1601986 10.1172/JCI115779 PMC295869

[32] Saisho Y, Butler a. E, Meier JJ, Monchamp T, Allen-Auerbach M, Rizza R a., et al Pancreas volumes in humans from birth to age one hundred taking into account sex, obesity, and presence of type-2 diabetes. Clin Anat. 2007;20: 933–942. doi: 10.1002/ca.20543 17879305 10.1002/ca.20543 PMC2680737

[33] Rodriguez-Calvo T, Ekwall O, Amirian N, Zapardiel-Gonzalo J, Von Herrath MG. Increased immune cell infiltration of the exocrine pancreas: A possible contribution to the pathogenesis of type 1 diabetes. Diabetes. 2014;63: 3880–3890. doi: 10.2337/db14-0549 24947367 10.2337/db14-0549 PMC4207385

[34] Skog O, Korsgren O. Aetiology of type 1 diabetes: Physiological growth in children affects disease progression. Diabetes Obes Metab. 2017; 1–11. doi: 10.1111/dom.13144 29083510 10.1111/dom.13144

