# Supplementary figures and images for: Decreased α-cell mass and early structural alterations of the exocrine pancreas in patients with type 1 diabetes: An analysis based on the nPOD repository
Source: PLoS One. 2018 Jan 19;13(1):e0191528. doi: 10.1371/journal.pone.0191528 (PMC5774815; doi:10.1371/journal.pone.0191528)

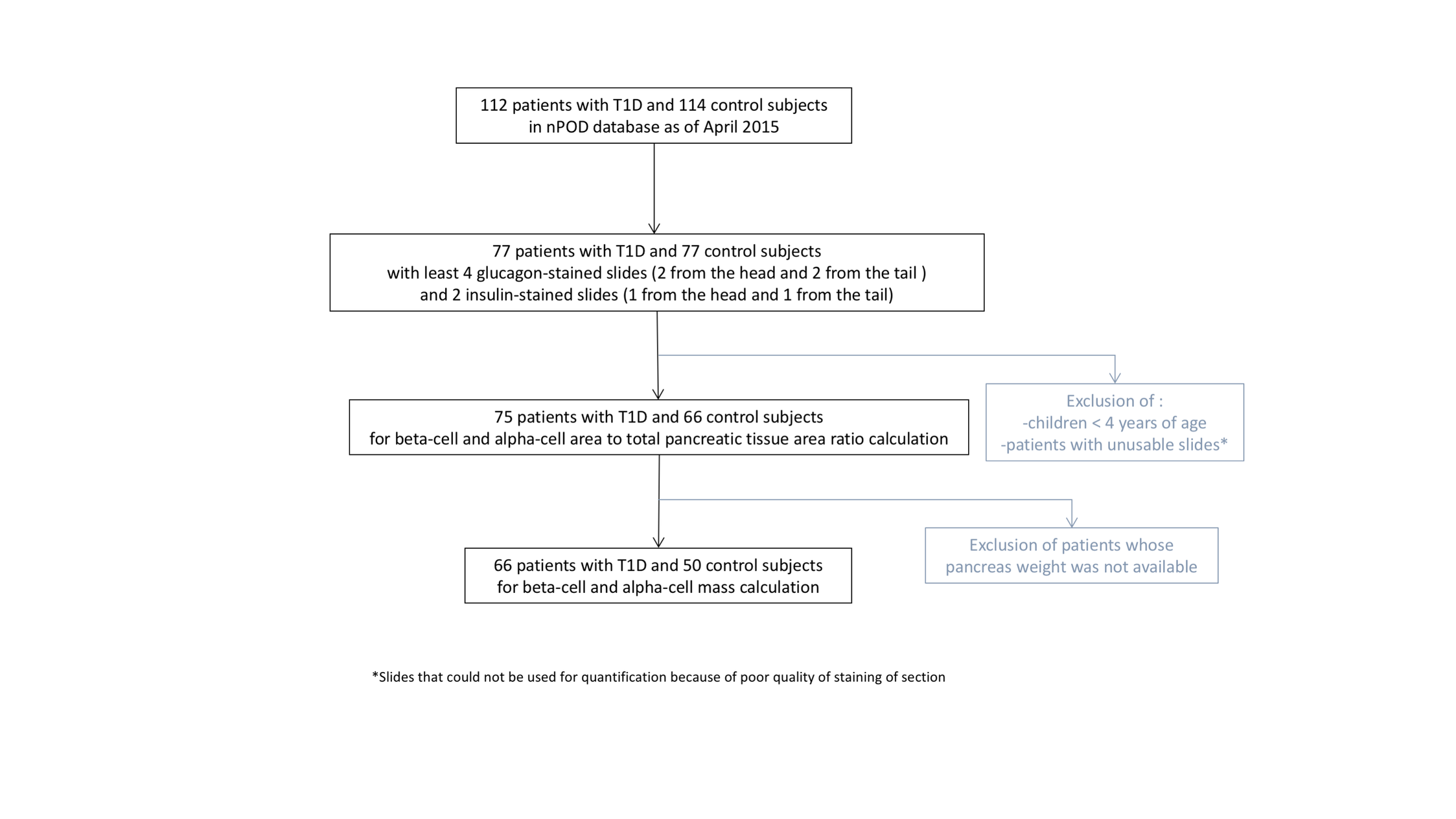

Supplement: S1 Fig — (TIF) [file pone.0191528.s001.tif]

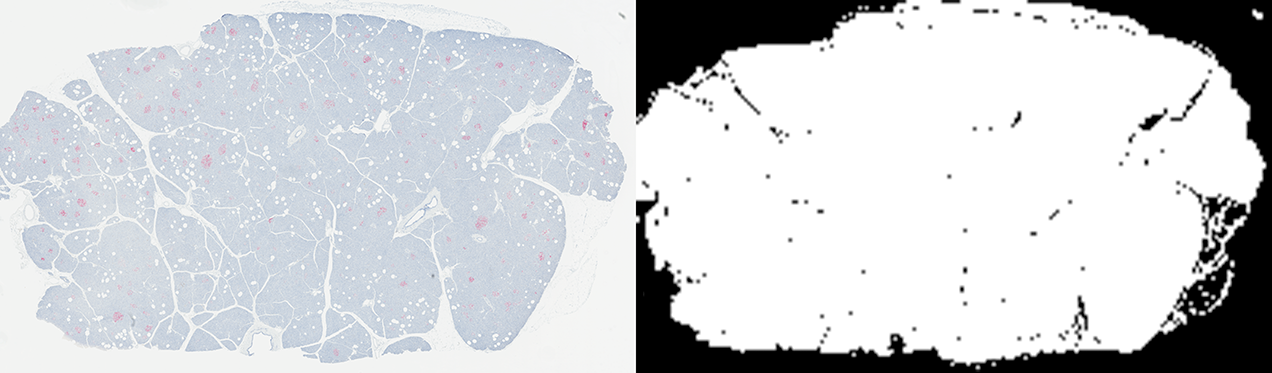

Supplement: S2 Fig — (TIF) [file pone.0191528.s002.tif]

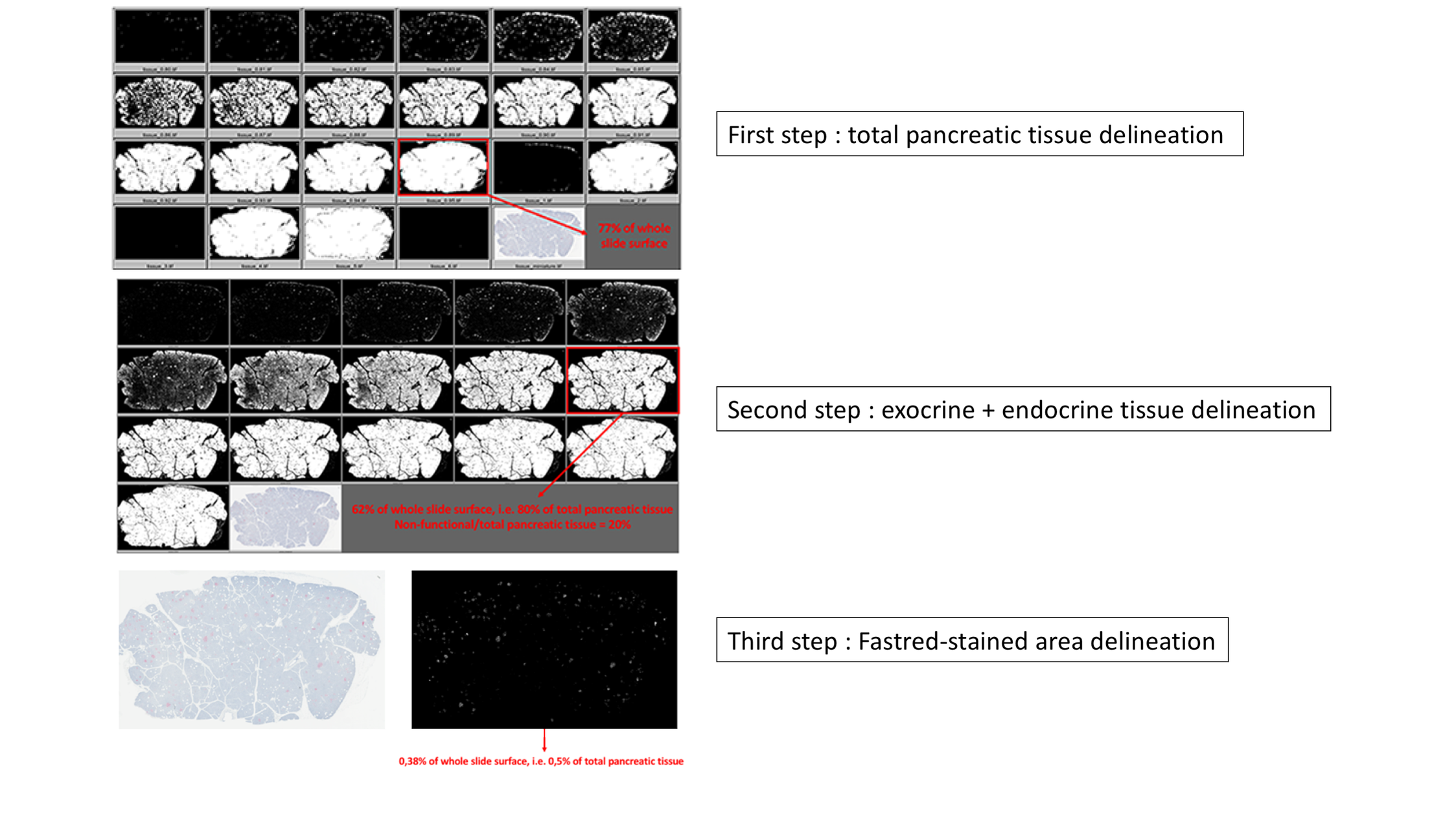

Supplement: S3 Fig — (TIF) [file pone.0191528.s003.tif]

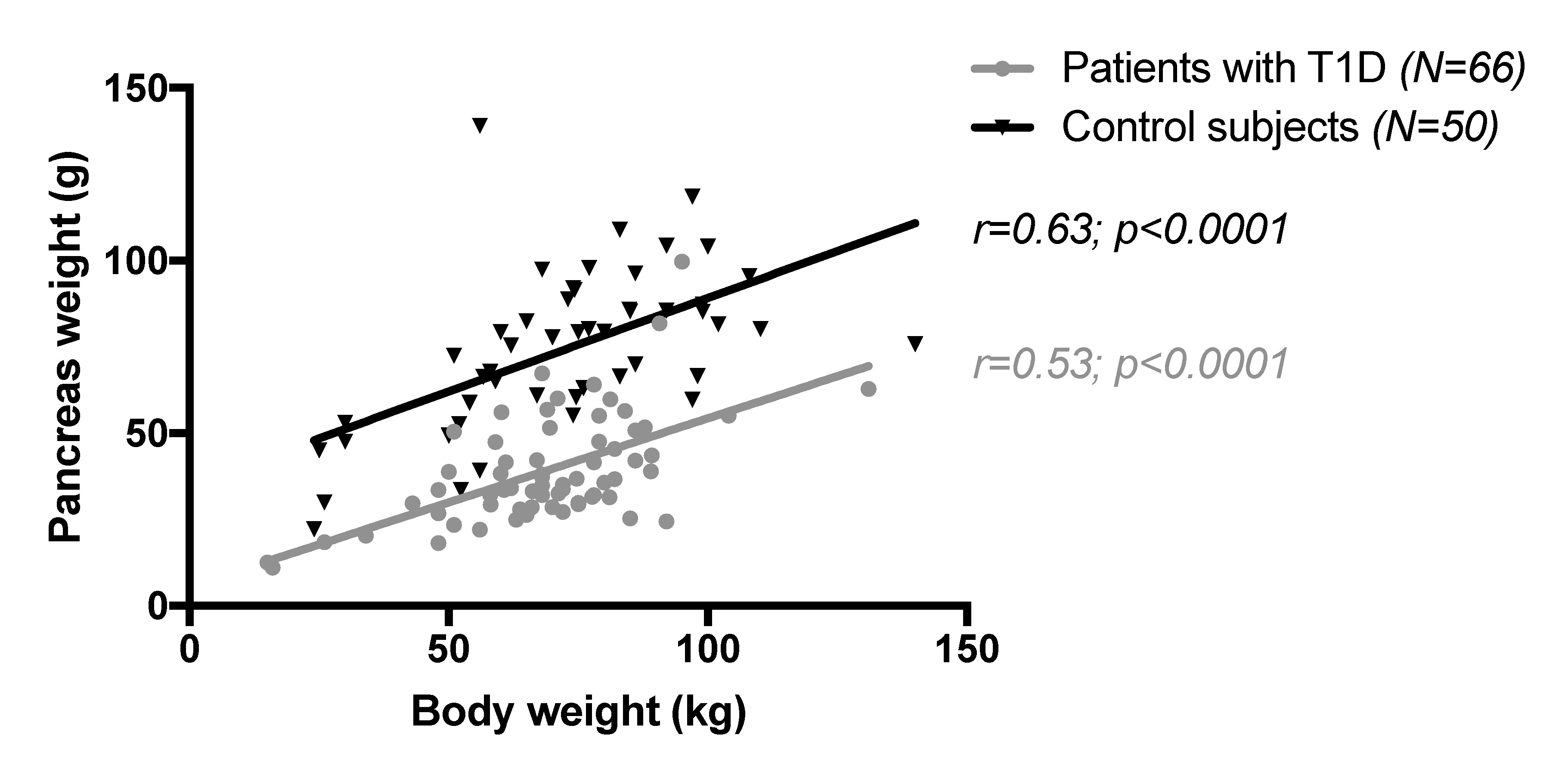

Supplement: S4 Fig — Data representation is the same as in Fig 6. (TIF) [file pone.0191528.s004.tif]

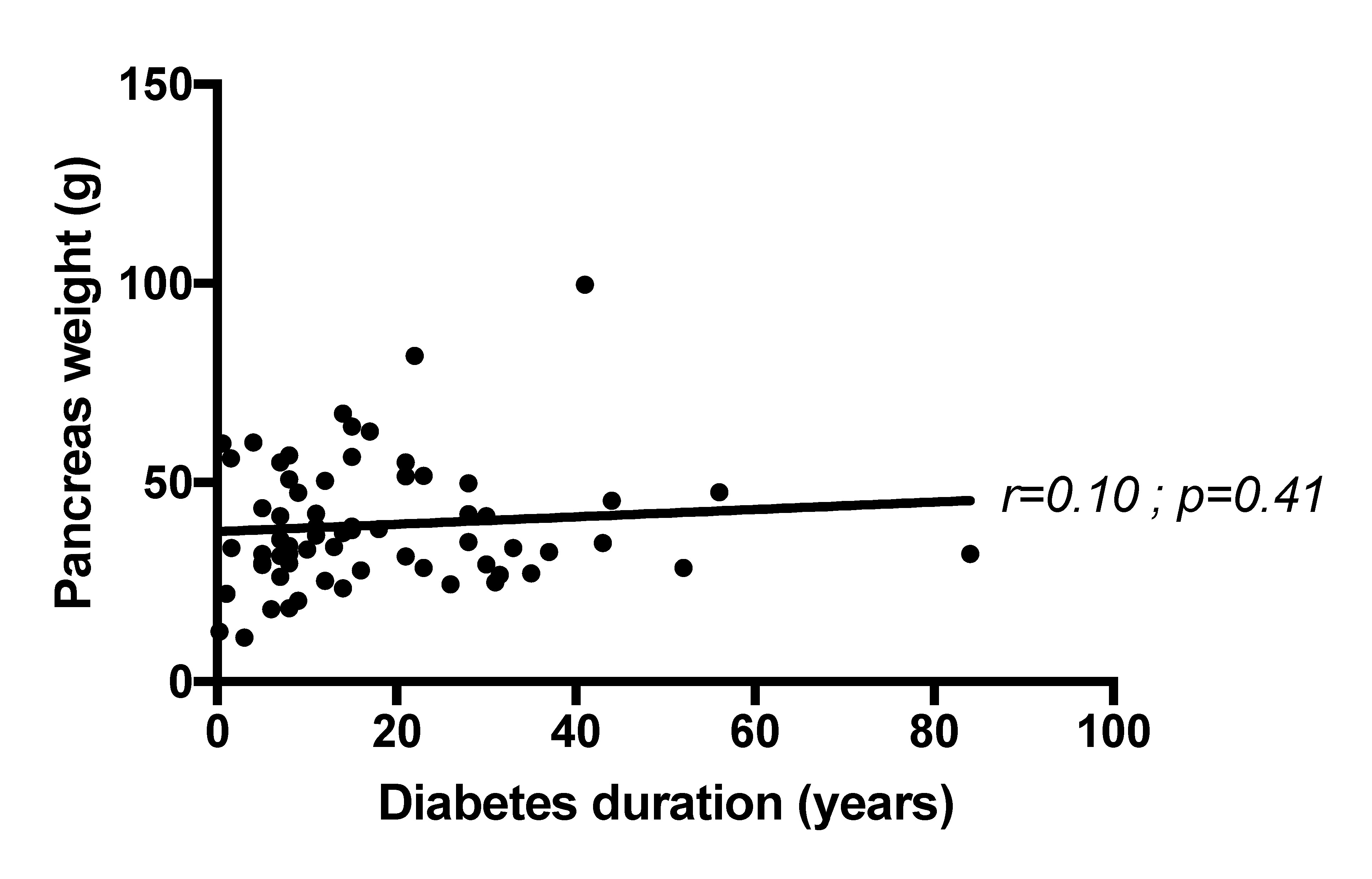

Supplement: S5 Fig — Data representation is the same as in Fig 6. (TIF) [file pone.0191528.s005.tif]

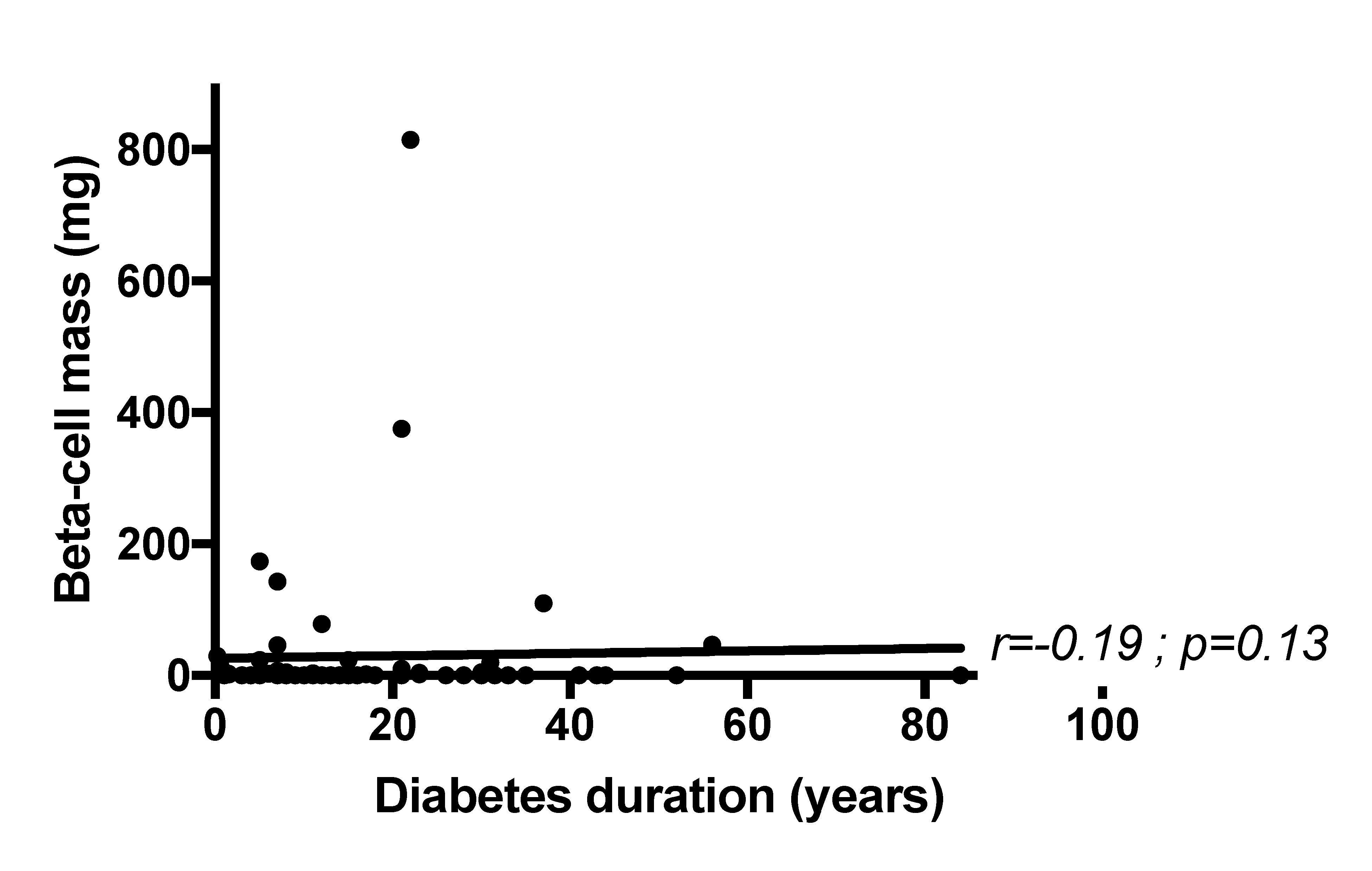

Supplement: S6 Fig — Data representation is the same as in Fig 6. (TIF) [file pone.0191528.s006.tif]

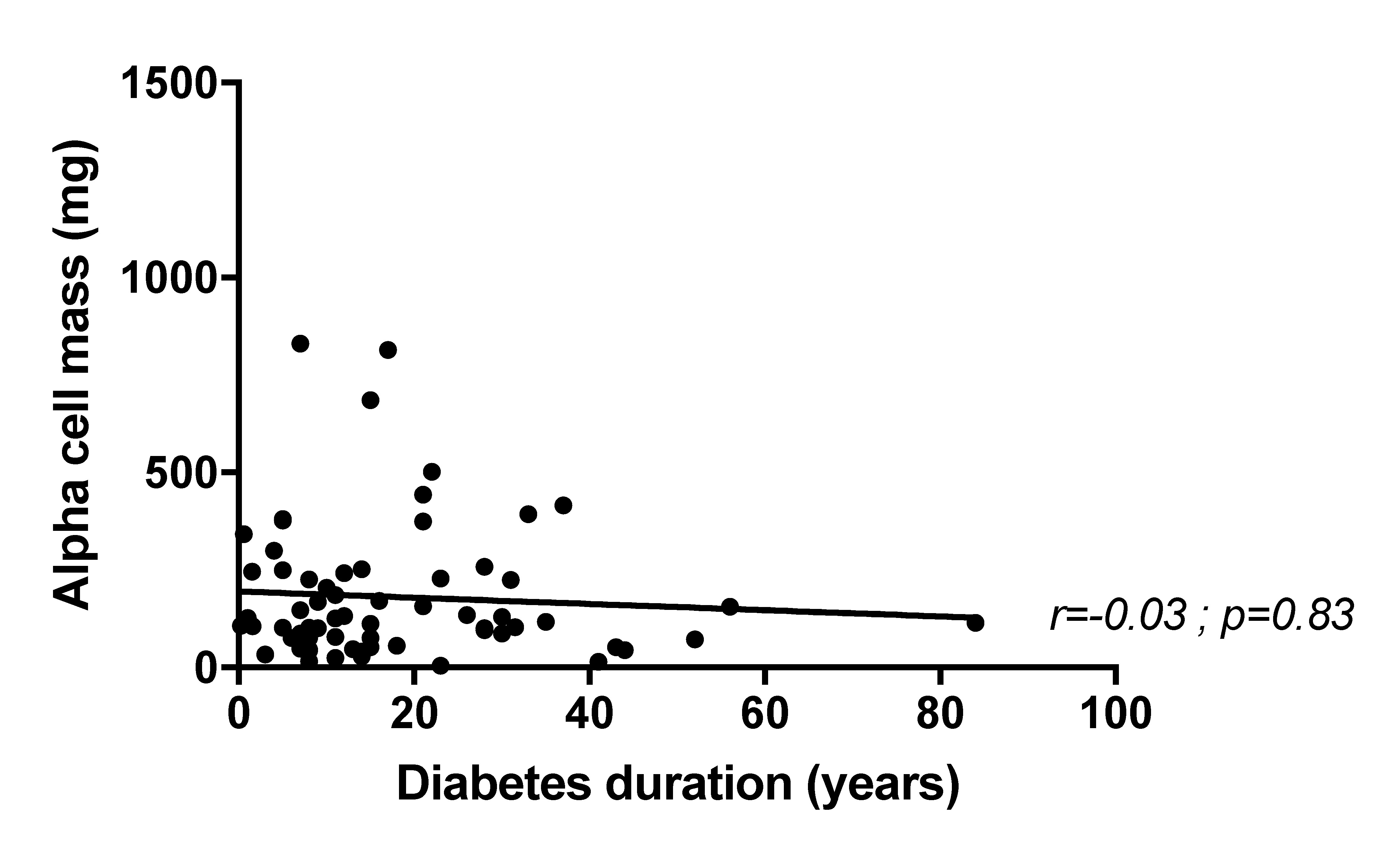

Supplement: S7 Fig — Data representation is the same as in Fig 6. (TIF) [file pone.0191528.s007.tif]

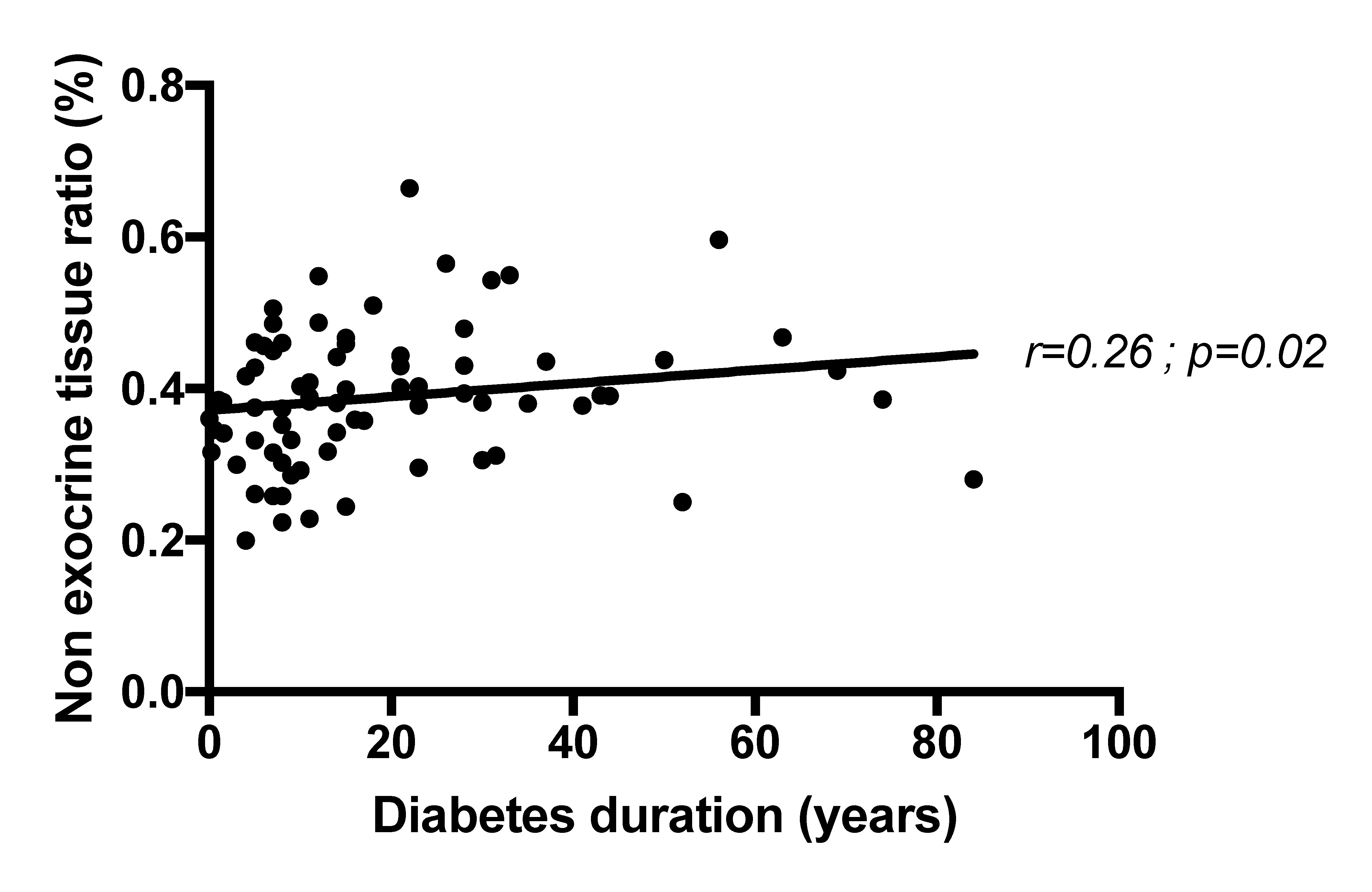

Supplement: S8 Fig — Data representation is the same as in Fig 6. (TIF) [file pone.0191528.s008.tif]
